# Elevated Fibroblast Growth Factor Signaling Is Critical for the Pathogenesis of the Dwarfism in *Evc2/Limbin* Mutant Mice

**DOI:** 10.1371/journal.pgen.1006510

**Published:** 2016-12-27

**Authors:** Honghao Zhang, Nobuhiro Kamiya, Takehito Tsuji, Haruko Takeda, Greg Scott, Sudha Rajderkar, Manas K. Ray, Yoshiyuki Mochida, Benjamin Allen, Veronique Lefebvre, Irene H. Hung, David M. Ornitz, Tetsuo Kunieda, Yuji Mishina

**Affiliations:** 1 Department of Biologic and Materials Sciences, School of Dentistry, University of Michigan, Michigan, United States of America; 2 Reproductive and Developmental Biology Laboratory (RDBL), National Institute of Environmental Health Sciences, National Institutes of Health, Research Triangle Park, Durham, North Carolina, United States of America; 3 Graduate School of Environmental and Life Science, Okayama University, Okayama City, Japan; 4 Knock Out Core, National Institute of Environmental Health Sciences, National Institutes of Health, Research Triangle Park, Durham, North Carolina, United States of America; 5 Department of Molecular and Cell Biology, Henry M. Goldman School of Dental Medicine, Boston University, Boston, Massachusetts, United States of America; 6 School of Medicine, University of Michigan, Michigan, United States of America; 7 Department of Cellular & Molecular Medicine, Cleveland Clinic Lerner Research Institute, Cleveland, Ohio, United States of America; 8 Department of Neurobiology and Anatomy, University of Utah School of Medicine, Salt Lake City, Utah, United States of America; 9 Department of Developmental Biology, Washington University School of Medicine, St. Louis, Missouri, United States of America; WUSTL, UNITED STATES

## Abstract

Ellis-van Creveld (EvC) syndrome is a skeletal dysplasia, characterized by short limbs, postaxial polydactyly, and dental abnormalities. EvC syndrome is also categorized as a ciliopathy because of ciliary localization of proteins encoded by the two causative genes, *EVC* and *EVC2* (aka LIMBIN). While recent studies demonstrated important roles for EVC/EVC2 in Hedgehog signaling, there is still little known about the pathophysiological mechanisms underlying the skeletal dysplasia features of EvC patients, and in particular why limb development is affected, but not other aspects of organogenesis that also require Hedgehog signaling. In this report, we comprehensively analyze limb skeletogenesis in *Evc2* mutant mice and in cell and tissue cultures derived from these mice. Both *in vivo* and *in vitro* data demonstrate elevated Fibroblast Growth Factor (FGF) signaling in *Evc2* mutant growth plates, in addition to compromised but not abrogated Hedgehog-PTHrP feedback loop. Elevation of FGF signaling, mainly due to increased *Fgf18* expression upon inactivation of *Evc2* in the perichondrium, critically contributes to the pathogenesis of limb dwarfism. The limb dwarfism phenotype is partially rescued by inactivation of one allele of *Fgf18* in the *Evc2* mutant mice. Taken together, our data uncover a novel pathogenic mechanism to understand limb dwarfism in patients with Ellis-van Creveld syndrome.

## Author Summary

Ellis-van Creveld (EvC) syndrome is a congenital skeleton disorder characterized by short limbs. Recent studies indicated that EVC and EVC2, the proteins encoded by two causative genes of EvC syndrome, play important function in transducing Hedgehog signaling, a signaling pathway critical for embryonic development. The defective Hedgehog signaling in chondrocytes is therefore the speculated reason for dwarfism in EvC patients. However, despite the apparent skeletal abnormalities observed in EvC patients, other tissues that require Hedgehog signaling are relatively normal. To understand how skeletal development is specifically affected in EvC syndrome, we analyze the limb skeletogenesis using *Evc2* mutant mice. Our data demonstrated that mutation in *Evc2* only moderately affected Hedgehog-PTHrP feedback loop in the growth plate, which only partially contributes to the dwarfism. Additionally, the elevated Fibroblast Growth Factor (FGF) signaling, another signal important for embryonic development, critically contributes to the pathogenesis of limb dwarfism. We identified that loss of EVC2 function in the perichondrium, the tissue surrounding growth plate chondrocytes, is critical to develop the dwarfism in the *Evc2* mutant mice. Overall, our data uncover a novel pathogenic mechanism to understand limb dwarfism in EvC patients and suggest that therapies for Achondroplasia caused by elevated FGF signaling may be applicable for relieving dwarfism found in EvC syndrome.

## Introduction

Ellis-van Creveld syndrome (EvC), a chondroectodermal dysplasia and mesoectodermal dysplasia, is an autosomal recessive congenital disease [1]. EvC patients generally bear a variety of defects such as shorter limbs and ribs, postaxial polydactyly, as well as dysplastic nails and teeth. Previous genetic studies have shown that more than two thirds of EvC patients carry mutations in either *EVC* or *EVC2* [2, 3]. Interestingly, *LIMBIN*, originally identified as the causative gene for chondrodysplastic dwarfism in Japanese Brown cattle, was later discovered as the cattle orthologue of *EVC2* [4]. A recent study reported spontaneous mutations in *EVC2* in Tyrolean Grey cattle [5]. In both cases, *EVC2* homozygous mutant cattle suffered from chondrodysplastic dwarfism, suggesting evolutionarily conserved functions of EVC2 during appendicular bone development.

Appendicular bone development occurs through endochondral ossification, a process during which a series of locally produced factors are interacting to ensure the correct length and shape of each skeletal element [6, 7]. Fibroblast Growth Factor (FGF) signaling, mediated by FGF18 produced in the perichondrium, is involved in this regulatory network [8, 9]. In the growth plate, FGF signaling inhibits chondrocyte proliferation through STAT1-mediated p21 expression [10, 11] and inhibits chondrocyte differentiation through MEK/pERK-mediated signaling [12]. In addition to FGF signaling, Indian Hedgehog and parathyroid hormone-related protein (PTHrP) signaling plays a central role in the regulation of chondrocyte proliferation, differentiation and maturation. Indian Hedgehog is synthesized by pre-hypertrophic chondrocytes [13] and stimulates PTHrP synthesis in chondrocytes at the distal end of the growth plate [14]. PTHrP promotes proliferation of chondrocytes and prevents them from progressing to pre-hypertrophic differentiation directly [14] and through suppression of FGF receptor 3 (*Fgfr3*) expression [15]. Once proliferating chondrocytes are far away enough from the source of PTHrP, they differentiate into pre-hypertrophic chondrocytes, start synthesizing Indian Hedgehog, and then further mature into hypertrophic cells.

Recently, primary cilium has been identified as a cellular organelle essential for Hedgehog signaling transduction [16]. Upon Hedgehog signaling induction, Smoothened (SMO) moves into the cilium [17]. At the same time, a protein complex consisting of glioma-associated oncogene (GLI) and Suppressor of Fused (SUFU) moves into the cilium, where it is quickly dissociated [18–20]. GLI proteins subsequently traffic out of the cilium and move into the nucleus, where they function as transcriptional activators of Hedgehog responsive genes [18]. Recent studies have also demonstrated that EVC and EVC2 interact with each other and are mutually required for localization at the base of the primary cilium [21–23]. Protein structure analysis coupled with biochemical analysis demonstrated that both EVC and EVC2 are N-terminus-anchored type I transmembrane proteins [24]. Despite that there is no functional domains identified in EVC or EVC2, they intracellularly localize at the base of the primary cilium through interaction with EFCAB7 and IQCE [25]. Upon induction of Hedgehog signaling, the EVC/EVC2 complex interacts with SMO at the base of cilium, which affects GLI2 accumulation in the ciliary tips [21, 23]. Consistent with *in vitro* mechanistic studies, diminished Hedgehog signaling was observed in the growth plates of mice lacking either *Evc* or *Evc2* [23, 26, 27]. Since the Hedgehog-PTHrP loop has a central role in regulating chondrocyte proliferation and maturation, thereby determining the length of the appendicular skeleton, disrupted Hedgehog signaling in the growth plate is the speculated reason for dwarfism in the EvC syndrome [21, 23].

Previous genetic studies have indicated that Hedgehog signaling plays critical roles during many processes during mouse embryonic development. For example, a loss-of-function mutation in *Shh* leads to holoprosencephaly, neural tube defect, and a variety of midline defects, such as cyclopia, cleft palate and cleft lips [28, 29], and a single digit and other limb skeletal defects [30]. A loss-of-function mutation in Indian Hedgehog (*Ihh*) was shown to severely impair endochondral ossification, resulting in extremely short and undermineralized limb bones [13] and in early closure of the growth plates [31]. Despite the indispensable functions implicated by previous studies for EVC/EVC2 in transducing Hedgehog signaling, except for short limbs, the aforementioned severe defects are observed neither in EvC patients nor in *Evc* and *Evc2* mutant mice. This discrepancy between the function of EVC/EVC2 in transducing Hedgehog signaling and phenotypic observations in *Evc* or *Evc2* mutant mice prompted us to ask to what extent does the loss of function of *Evc/Evc2* reduce Hedgehog signaling, and why does *Evc/Evc2* loss of function specifically impact limb development. To answer these questions, we investigated Hedgehog signaling and FGF signaling levels during defective endochondral ossification in *Evc2* mutant mouse lines [27]. We demonstrate that a nonsense mutation in *Evc2* that mimics mutations seen in EvC patients, leads to compromised but not abrogated Hedgehog signaling. In addition, we found that FGF signaling is significantly elevated in the *Evc2* mutant growth plate due to increased expression of *Fgf18* in the perichondrium. We successfully demonstrated that inactivation of one allele of *Fgf18* in the *Evc2* mutant mice partially rescued the dwarfism phenotype. We conclude that both reduced Hedgehog signaling and elevated FGF signaling play a critical role in the pathogenesis of the unique form of dwarfism that characterizes *Evc2* mutants. Our findings explain differences between proposed functions of EVC/EVC2 based on biochemical approaches and symptoms found in the EvC patients, and thus provide insight for better options to treat dwarfism found in EvC patients.

## Results

### *Evc2* mutation affects chondrocyte proliferation and maturation

**Fig 1:**
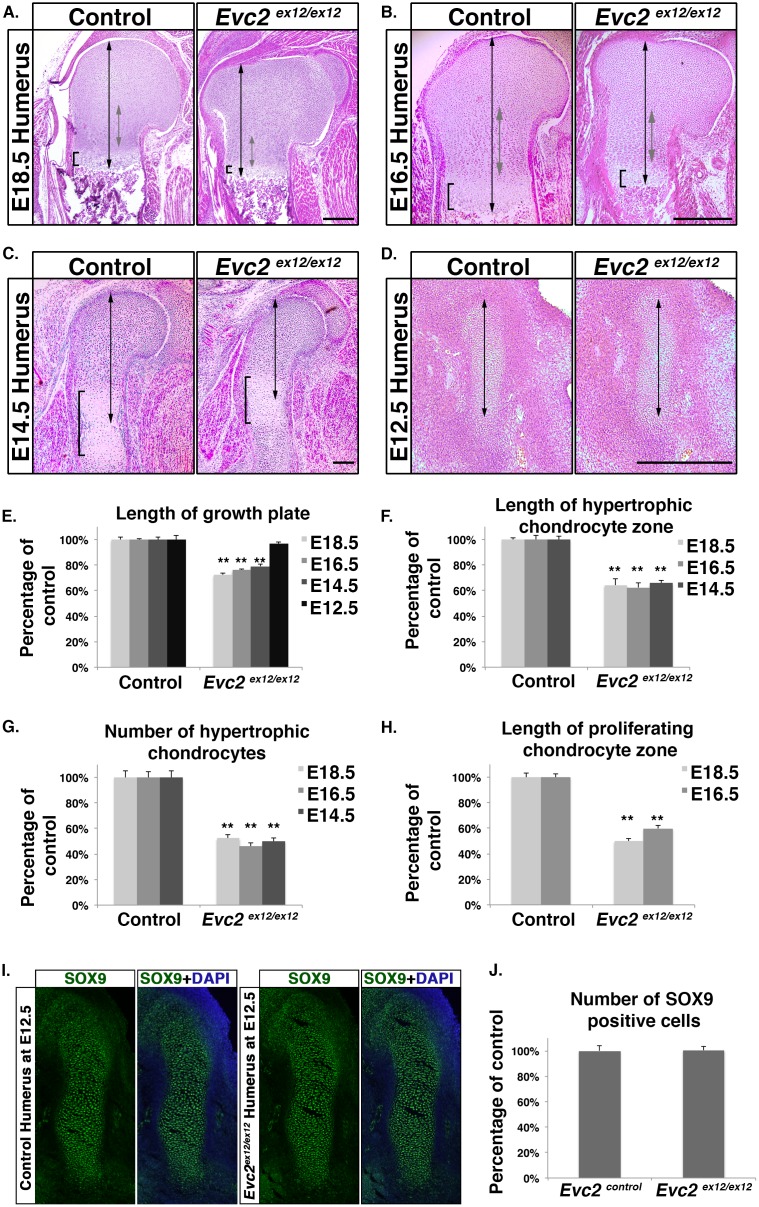
A homozygous inactivating mutation in *Evc2* leads to defective limb growth. H and E staining of humerus proximal growth plates from control and *Evc2* mutant embryos at E18.5 (A), E16.5 (B), E14.5 (C) and cartilage primordia at E12.5 (D). The black double arrows indicate the length of the proximal growth plates (A-C), and the entire cartilage primordia (D). At E14.5, since a bone tissue has not separated the presumptive proximal growth plate and the presumptive distal growth plate, we take measurements from the top of the cartilage primordia to the center of the hypertrophic chondrocyte area (C) as lengths of the proximal growth plates. The grey double arrows indicate the length of the proliferating chondrocyte zone (A, B). The brackets indicate the hypertrophic chondrocyte zone of the proximal growth plate (A, B) and the area of hypertrophic chondrocytes at the middle of the cartilage primordia (C). Quantifications of the growth plate lengths (E), hypertrophic chondrocyte zone lengths (F), hypertrophic chondrocyte cell numbers (G) and proliferating chondrocyte zone lengths (H) at the indicated stages are shown. The percentages of the control are shown in each chart. Immunohistochemistry of SOX9 of humerus at E12.5 (I) indicates the condensed mesenchyme in humerus. Quantifications of the length of SOX9 positive areas (J) are shown. (Error bars indicate standard errors. (n = 6, ** p<0.01). Scale bars: 200 μm for (A), (B), (C) and (D).

We generated *Evc2* mutant mice by introducing a premature stop codon, along with an IRES-LacZ cassette, into exon12 (equivalent to human exon 14) to mimic one of the nonsense mutations identified in human patients [32]. Compared with control littermates, homozygous mutant mice showed a decrease in body length as well as in appendicular bone length at 4 weeks of age (S1A Fig). They did not show a difference in body length or body weight at birth (S1B and S1C Fig), but they already had shorter limb bones [27]. *Evc2* mutant mice were thus born with disproportionately short limbs. Staining of heterozygous mutant tibia growth plates for β-Gal activity indicated that *Evc2* was expressed in chondrocytes as well as the perichondrium (S1D Fig), which is consistent with RNA *in situ* hybridization results [4]. Histological analysis of humeral growth plates indicated shorter hypertrophic and proliferating chondrocyte zones, and fewer hypertrophic chondrocytes in *Evc2* mutant embryos at E18.5 (Fig 1A, 1E, 1F, 1G and 1H). Similar characteristics were observed at E16.5 (Fig 1B, 1E, 1F, 1G and 1H), and E14.5 (Fig 1C, 1E, 1F and 1G). On the other hand, histologic analysis at E12.5, when chondrocytes were just starting to differentiate from condensed mesenchyme, showed no difference in the length of cartilage primordia (Fig 1D, 1I and 1J). The same tendency was also observed in other limb skeletal elements, such as ulna, radius, femur and tibia [27]. These observations suggest that mutation of *Evc2* leads to dwarfism by affecting growth plate chondrocyte proliferation and/or maturation, but not by affecting mesenchymal condensation or differentiation of condensed mesenchymal cells into chondrocytes.

### Hedgehog signaling is compromised but not abrogated in *Evc2* mutant growth plates

It has been reported that EVC2 is a ciliary protein [24, 26]. To examine whether *Evc2* mutation leads to loss of ciliary EVC2, we visualized EVC2 protein in embryonic growth plates using an antibody recognizing the N-terminus of EVC2. As expected, EVC2 was localized at the base of the cilia in control growth plates, but was undetectable in cilia of *Evc2* mutant growth plates (S2A and S2B Fig). Similarly, our previous work [27] indicated that *Evc2* mutant primary chondrocytes do not have ciliary EVC2 or EVC. These findings strongly suggest that the truncation mutation at exon12 of *Evc2* leads to abrogation of ciliary localization of both EVC2 and its interaction partner EVC.

**Fig 2:**
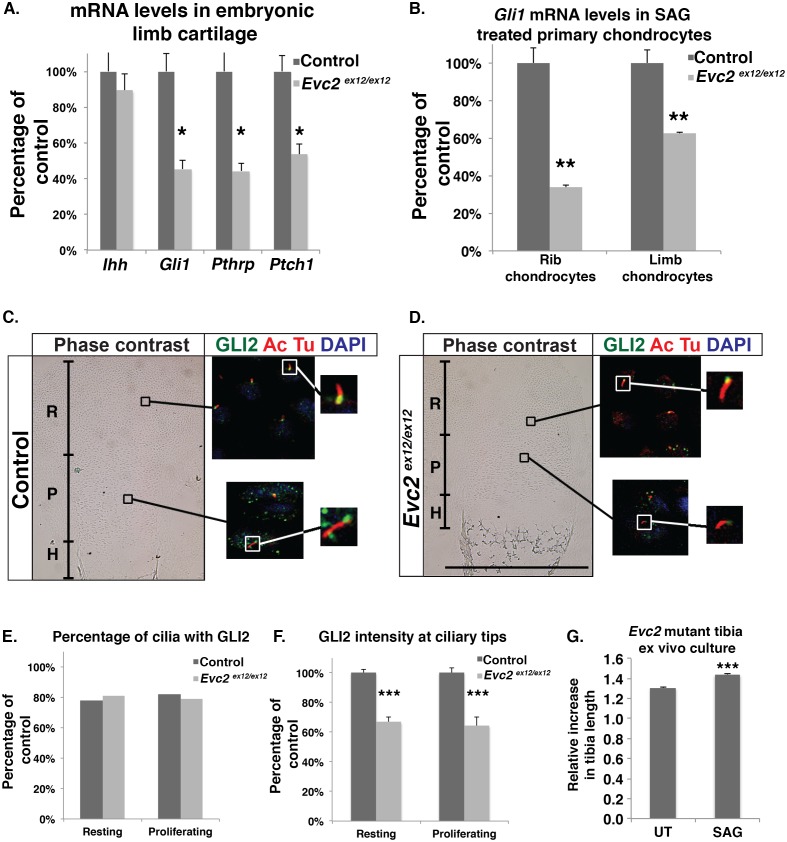
Compromised but not abrogated Hedgehog signaling is detected in *Evc2* mutant mice. A. Q-RT-PCR quantification of mRNA levels for the indicated genes in tibial cartilage from E16.5 embryos (n = 6, * p<0.05). B. Fold changes in Hedgehog signaling in primary chondrocytes isolated from E18.5 ribs and knee joints. Cell cycle was arrested before treatment with 100 nM SAG. Fold changes of *Gli1* mRNA levels due to treatment are presented relative to control levels before treatment (n = 3, **p<0.01). C-D. E16.5 humerus growth plates from control embryos (C) and *Evc2* mutant littermates (D) were stained for acetylated tubulin (ciliary marker, red), GLI2 (green) and DNA (blue). R: Resting chondrocyte, P: Proliferating chondrocyte, H: Hypertrophic chondrocyte. E. Quantification of the percentage of cilia positive for GLI2 staining at the tip in control and mutant samples shown in panel C and D (n = 80). F. Quantification of the intensity of GLI2 staining at ciliary tips in control and mutant samples (n = 40, p<0.001). G. Tibia *ex vivo* culture indicated that *Evc2* mutants responded to SAG treatment. Tibiae were dissected out from E16.5 *Evc2* mutant hindlimbs and cultured with or without 1 μM SAG for 7 days. Growth rates were calculated using the tibia length at D7 compared to D0 (n = 5, *** p<0.001). UT, untreated. Scale bars: 200 μm for (C) and (D).

Hedgehog signaling, mediated by Indian Hedgehog in the growth plate, plays an important role in directing chondrocyte proliferation and hypertrophic maturation. Consistent with previous studies in *Evc* and *Evc2* mutant mice [23, 26], we also detected decreased Hedgehog signaling in *Evc2* mutant growth plates [27]. To evaluate the remaining Hedgehog signaling levels, we dissected out E16.5 tibia cartilage from control and *Evc2* mutant growth plates for RNA isolation. qRT-PCR for *Gli1*, *Ptch1* and *Pthrp* (Fig 2A), which are direct targets of Hedgehog signaling, indicated that Hedgehog signaling was significantly reduced, up to 40% of its normal level, in *Evc2* mutants. These observations are consistent with our previous report on the Hedgehog signaling level using Gli1-lacZ reporter mice [27]. On the other hand, *Ihh* expression in *Evc2* mutant growth plates remained at the control level (Fig 2A). The results from in situ hybridization were consistent with expression analysis from qRT-PCR. Despite increased signal intensity of *Ihh* expression detected in *Evc2* mutants, the expression area is more restricted in *Evc2* mutant growth plate (S3A Fig), which is consistent with previous reports [23, 26]. To test whether this reduced Hedgehog signaling reflected impaired response of *Evc2* mutant chondrocytes to the Hedgehog ligand, we isolated primary chondrocytes and examined their response to Hedgehog signaling induction by a smoothened agonist (SAG). Similarly to what we observed *in vivo*, quantification of *Gli1* mRNA levels indicated that Hedgehog signaling was reduced to 60% and 40% in SAG-treated limb and rib primary chondrocytes from *Evc2* mutant mice, respectively, compared to SAG-treated control cells (Fig 2B). The accumulation of GLI proteins in ciliary tips is regarded as a hallmark of Hedgehog signaling induction [18]. To analyze the ciliary localization of GLI2, we co-stained E16.5 tibia growth plates for GLI2 with acetylated tubulin, a ciliary marker. In contrast with previous observations in cultured cells [21], we found decreased GLI2 accumulation in ciliary tips in both resting and proliferating chondrocytes of *Evc2* mutant growth plates (Fig 2C, 2D and 2F), while the percentage of cilia with GLI2 staining remained the same in control and *Evc2* mutants (Fig 2E). Induction of Hedgehog signaling in primary chondrocytes also resulted in diminished accumulation of GLI2 (S4A Fig), SUFU (S4C Fig) and KIF7 (S4E Fig) in ciliary tips, with no effect on ciliary accumulation of SMO (S4G Fig). To further confirm that tissues/cells bearing *Evc2* mutation can still respond to Hedgehog signaling to some extent, we treated *Evc2* mutant tibiae with the Smoothened agonist, SAG, *ex vivo* and observed significant increases in tibia length compared with untreated *Evc2* mutant tibiae (Fig 2H). All aforementioned data thus concurred that the mutation of *Evc2* leads to compromised but not abrogated Hedgehog signaling, likely due to impaired accumulation of Hedgehog components in ciliary tips.

### *Evc2* mutant growth plates display elevated FGF signaling

**Fig 3:**
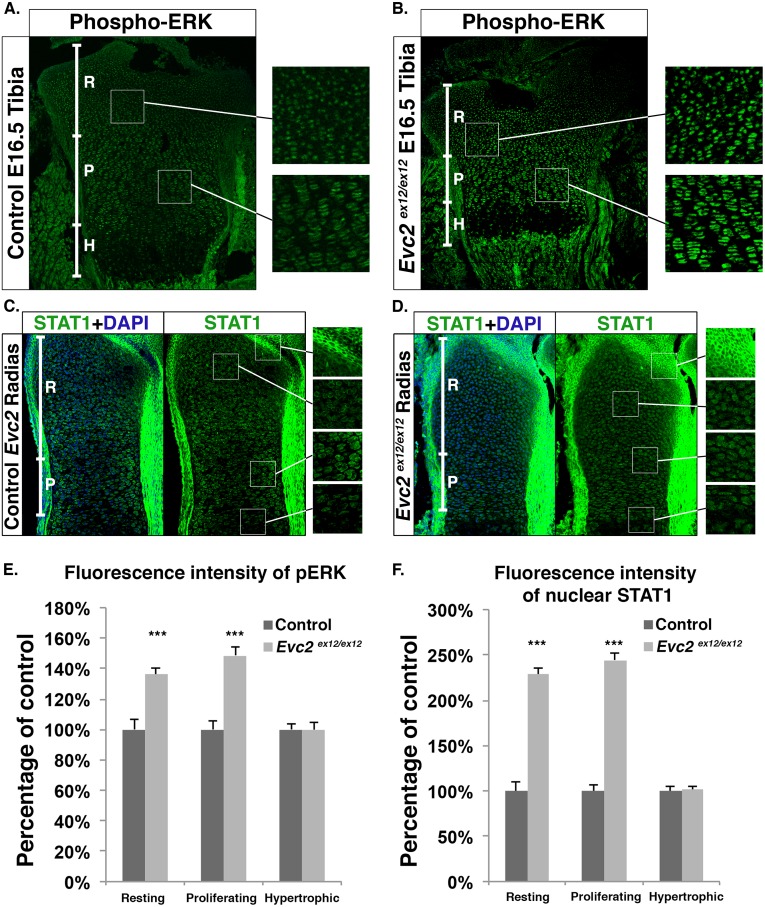
Increased FGF signaling is detected in *Evc2* mutant growth plates. A-B. Immunohistochemistry for phospho-ERK in tibia proximal growth plates from E16.5 control (A) and mutant (B) littermates. C-D. Immunohistochemistry of nuclear STAT1 in radius growth plates of E16.5 control (D) and mutant littermates (E). In all four panels, enlarged areas of distinct growth plate zones are shown on the right. E-F. Quantification of phospho-ERK (E) and nuclear STAT1 (F) signal intensities. Data are presented as percentages of controls (n = 80, ***p<0.001). R: Resting chondrocytes, P: Proliferating chondrocytes, H: Hypertrophic chondrocytes.

We sought to further dissect the mechanisms by which *Evc2* loss of function specifically impacts limb development. The aforementioned decrease in length of the hypertrophic chondrocyte zones in *Evc2* mutants is characteristic of Achondroplasia, the most common form of dwarfism in humans caused by gain of function mutations in FGF receptor 3 (FGFR3) [33, 34] To examine whether *Evc2* mutant growth plates have altered FGF signaling, we first examined their phospho-ERK level by immunohistochemistry. In E16.5 *Evc2* mutant tibiae, we detected elevated phospho-ERK in both resting and proliferating chondrocytes, but not in hypertrophic chondrocytes (Fig 3A and 3B). Quantification indicated an increase of about 50% in *Evc2* mutant growth plates compared to controls (Fig 3E). Beside elevating ERK phosphorylation, FGF signaling is also known to slow down cell cycle progression through STAT1-mediated p21 expression [10, 11]. Immunofluorescence for STAT1 demonstrated less plasma membrane-associated and more nuclear STAT1 in resting and proliferating chondrocytes in *Evc2* radii than in controls (Fig 3C, 3D and 3F). On the other hand, as a negative control, in the perichondrium, there was no nuclear STAT1 detected in *Evc2* mutants, which is consistent with a previous report that only FGFR3 (expressed in resting and proliferating chondrocytes) but not FGFR2 (expressed in perichondrium) can induce STAT1 nuclear translocation [10].

**Fig 4:**
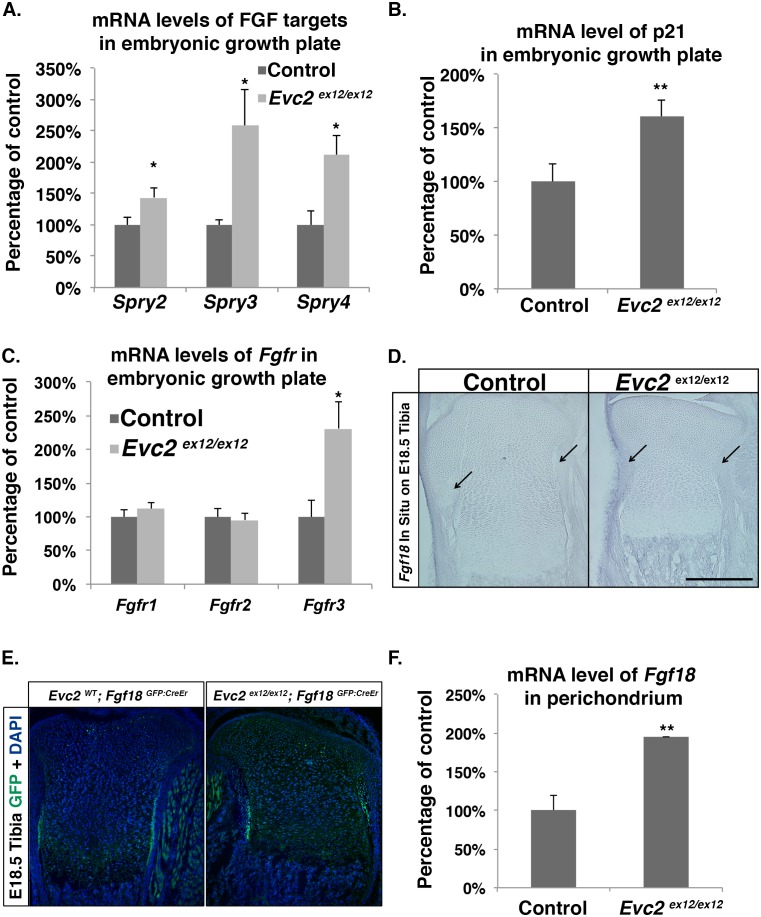
Growth plates in *Evc2* mutants have elevated FGF signaling. A. qRT-PCR assay of *Spry2*, *Spry3* and *Spry4* mRNA levels in growth plate cartilage from E16.5 *Evc2* mutant and control littermates (n = 6, *p<0.05). B. qRT-PCR assay of *p21* mRNA levels in the same samples as in panel A. C. qRT-PCR assay of *Fgfr1*, *Fgfr2* and *Fgfr3* mRNA levels in the same samples as in panel A. D. *In situ* hybridization indicates elevated *Fgf18* RNA levels in the perichondrium of tibiae from E18.5 *Evc2* mutants. Arrows indicate the perichondrium in both controls and mutants. E. Immunohistochemistry of GFP in the proximal tibie in *Evc2*^*WT*^; *Fgf18*^*GFP*:*CreEr*^ and *Evc2* ^*ex12/ex12*^; *Fgf18*^*GFP*:*CreEr*^ indicates an elevated *Fgf18* expression in *Evc2* mutants. F. qRT-PCR assay of *Fgf18* mRNA levels in the perichondrium of E16.5 control and *Evc2* mutant tibiae (n = 4, p<0.01). Scale bars: 200 μm for (D).

To confirm the elevation of FGF signaling in *Evc2* mutants, we examined the expression of FGF signaling targets in the growth plate. *Spry2*, *Spry3*, and *Spry4* were all significantly overexpressed in *Evc2* mutant growth plates (Fig 4A). An increase of *Spry3* expression was also detected in the embryonic limbs by *in situ* hybridization (S5A Fig). It is known that in chondrocytes, STAT1 activation up-regulates p21 expression [10, 11]. Consistently, we detected an elevated level of p21 mRNA in embryonic growth plate cartilage in *Evc2* mutants (Fig 4B). In conclusion, the mutation of *Evc2* led to an elevation of FGF signaling that is likely to contribute to the dwarfism phenotype.

### Increased expression of *Fgfr3* in chondrocytes and *Fgf18* in perichondrium likely contributes to the elevation of FGF signaling in *Evc2* mutants

To dissect the mechanism leading to elevated FGF signaling in *Evc2* mutants, we first evaluated the expression levels of FGF receptors 1, 2 and 3 in the growth plate. Consistent with our observation that elevated FGF signaling was only detected in resting and proliferating chondrocytes, we detected elevated expression for *Fgfr3* (Fig 4C), which is exclusively expressed in resting and proliferating chondrocytes [35], but did not detect any expression change for *Fgfr2*, which is specifically expressed in perichondrium [36] or *Fgfr1*, primarily expressed in osteoblasts [36]. The increased *Fgfr3* expression is also supported by the *in situ* hybridization of *Fgfr3* in the embryonic limbs (S5B Fig). It was previously observed that PTH/PTHrP treatment could repress *Fgfr3* expression in a chondrocyte cell line *in vitro* [37] and in growth plate chondrocytes *in vivo* [15]. As a direct target of Hedgehog signaling in the growth plate, *Pthrp* expression was decreased in *Evc2* mutants (Fig 2A), an effect that may lead to derepression of *Fgfr3* expression. To test this potential regulatory network, we evaluated the impact of PTH on the expression of *Fgfr3* in wild type primary chondrocytes. PTH (1–34) binding to the PTH/PTHrP receptor can elicit downstream signaling as a substitute for PTHrP in chondrocytes [38]. Treatment of primary chondrocytes with PTH for 24 h led to a 75% decrease in *Fgfr3* expression (S6 Fig). These results thus suggest that elevation of *Fgfr3* expression, occurring as a secondary effect of compromised Hedgehog signaling, may contribute to the increase in FGF signaling observed in *Evc2* mutant growth plates. FGF18, an FGF ligand documented to regulate endochondral ossification, is produced by the perichondrium surrounding the growth plates [8, 9, 39–41]. To assess *Fgf18* expression in *Evc2* mutants, we isolated perichondrium and detected a two-fold increase in *Fgf18* mRNA level in *Evc2* mutants compared to controls (Fig 4F). On the other hand, we did not detect changes in the expression levels of *Fgf9* or *Fgf23*, FGFs that also regulate skeletal development [41, 42]. Elevated *Fgf18* expression in the perichondrium was corroborated by *Fgf18* RNA *in situ* hybridization in tibia growth plates and immunohistochemistry of GFP in *Evc2* mutant carrying an *Fgf18*^*GFP*:*CreEr*^ allele (Fig 4D and 4E). Taken together, our data suggest that both elevated *Fgfr3* expression, as a secondary effect of compromised Hedgehog signaling, and elevated *Fgf18* expression in the perichondrium contribute to elevating FGF signaling in *Evc2* mutant growth plates.

### Tibia *ex vivo* culture suggests elevated FGF signaling in *Evc2* mutant growth plates

**Fig 5:**
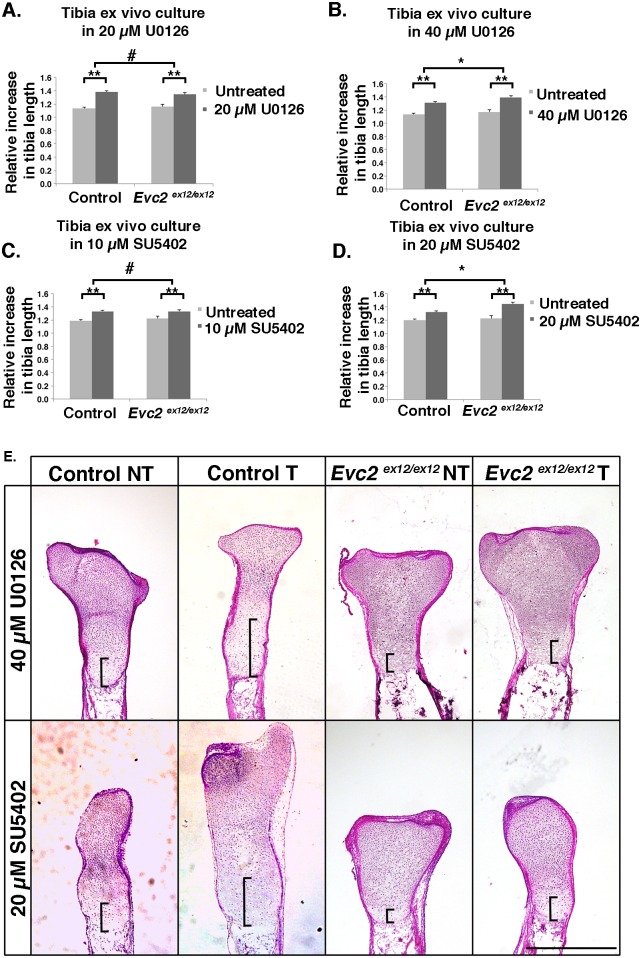
Tibia *ex vivo* culture indicates elevated endogenous FGF signaling in *Evc2* mutant growth plates. E16.5 control or *Evc2* mutant tibiae were cultured with 20 μM (A) or 40 μM (B) U0126, or with 10 μM (C) or 20 μM (D) SU5402 for 7 days. Growth rates were calculated as the tibia length at D7 relative to D0 (n = 6, * p<0.05, ** p<0.01, # p>0.2). (E) H&E staining of tibiae after *ex vivo* culture. (NT, no treatment; T, treatment). Pictures show the proximal half of tibiae, with brackets pointing to drastic differences in the height of the hypertrophic chondrocyte zone among samples. Scale bars: 200 μm for (E).

To examine if increased endogenous FGF signaling in *Evc2* mutant growth plates is a potential cause of the dwarfism phenotype, we set up tibia *ex vivo* cultures and promoted bone growth by suppressing endogenous FGF signaling. Tibiae from control and *Evc2* mutants grew at similar rates during a 7-day culture without FGF signaling inhibitor treatment (1.13±0.03 and 1.16±0.03, Fig 5A). At a low concentration of U0126 (20 μM), an inhibitor of MEK (activated by FGFR and activator of ERK), tibiae from control and mutant mice grew faster than untreated samples (1.38±0.03 and 1.34±0.03, respectively, p<0.01, Fig 5A), but there was no statistically significant difference between the two genotypes (Fig 5A, #). In contrast, at a higher concentration of U0126 (40 μM), *Evc2* mutant tibiae grew faster (1.39±0.02) than controls (1.31±0.02) relative to untreated samples (Fig 5B, *, p<0.05). Similar growth patterns were observed when SU5402, a specific inhibitor for FGF receptor kinase activity, was applied (10 and 20 μM, Fig 5C and 5D). Histologic analysis indicated that in both control and *Evc2* mutant tibiae (Fig 5E), the U0126 and SU5402 treatments led to an increase in the length of growth plates and hypertrophic chondrocyte zones. These data thus suggest that higher levels of endogenous FGF signaling in *Evc2* mutant growth plates contribute to the dwarfism in *Evc2* mutants.

### Compromised Hedgehog signaling partially contributes to the pathogenesis of dwarfism in *Evc2* mutants

**Fig 6:**
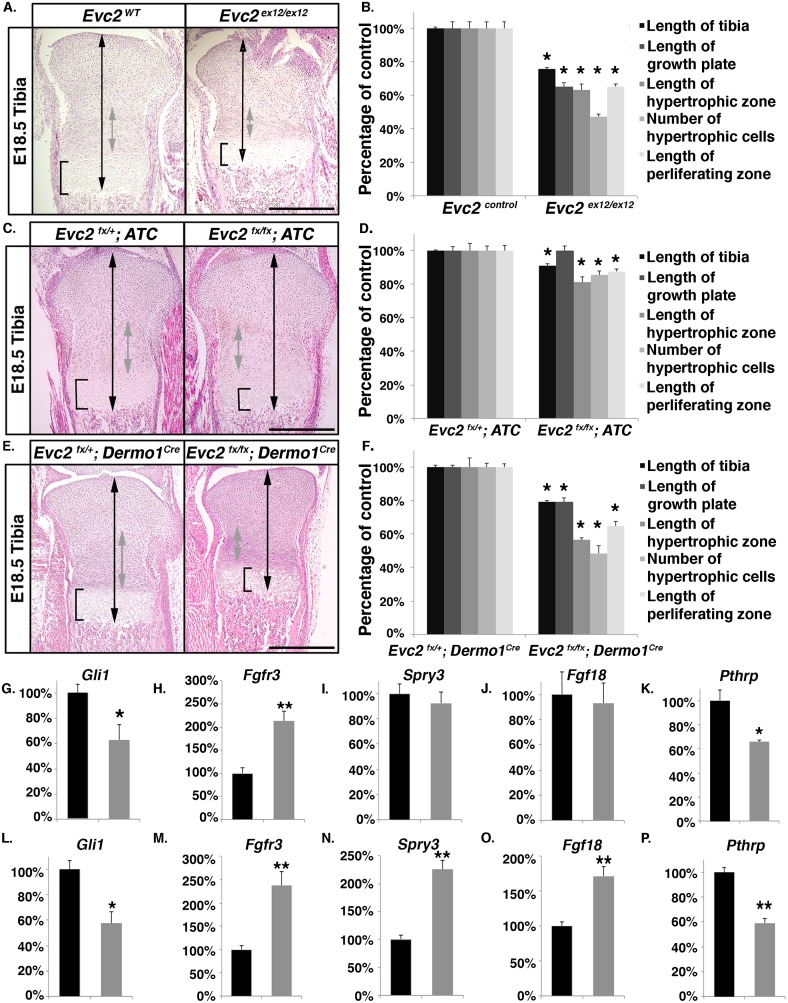
Elevated FGF signaling plays critical role in the pathogenesis of dwarfism in *Evc2* mutants. A. H&E staining of tibia proximal growth plates from E18.5 *Evc2* mutants and littermate controls. B. Quantification of growth plate zone lengths from similar samples as in panel A (n = 6, * p<0.01). C. H&E staining of tibia proximal growth plates from E18.5 embryos with *ATC*-mediated *Evc2* deletion and from littermate controls. D. Quantification of growth plate zone lengths from similar samples as in panel C (n = 6, * p<0.01). E. H&E staining of tibia proximal growth plates from E18.5 embryos with *Dermo1*^*Cre*^-mediated *Evc2* deletion and from littermate controls. F. Quantification of growth plate zone lengths from similar samples as in panel E (n = 6, * p<0.01). G-P. qRT-PCR assays of mRNA levels of indicated genes. Total RNA was isolated from the growth plates of (G-K) embryos with *ATC-*mediated *Evc2* deletion (*Evc2* ^*fx/fx*^; *ATC*, grey bar) and littermate controls (*Evc2* ^*fx/+*^; *ATC*, black bar), and (L-P) embryos with *Dermo1*^*Cre*^*-*mediated *Evc2* deletion (*Evc2* ^*fx/fx*^; *Dermo1*^*Cre*^, grey bar) and littermate controls (*Evc2* ^*fx/+*^; *Dermo1*^*Cre*^, black bar) (n = 5, *p<0.05, ** p<0.01). J. Expression levels of *Fgf18* were evaluated by qRT-PCR using RNA isolated from the perichondrium in *ATC* mediated *Evc2* mutant and littermate control (n = 5). Expression levels of *Gli1* (K), *Fgfr3* (L) and *Spry3* (M) were evaluated by qRT-PCR using RNA isolated from the growth plate cartilage in *Dermo1*^*Cre*^ mediated *Evc2* mutant and littermate control (n = 5, *p<0.05, ** p<0.01). O. Expression levels of *Fgf18* were evaluated by qRT-PCR using RNA isolated from the perichondrium in *Dermo1*^*Cre*^ mediated *Evc2* mutant and littermate control (n = 5, ** p<0.01). Scale bars: 200 μm for (A), (C) and (E).

Taken together, all results presented so far suggest that in addition to compromised Hedgehog signaling, elevated FGF signaling also contributes to the shortening of *Evc2* mutant growth plates. In *Evc2* mutants, compromised Hedgehog signaling is possibly due to impaired ciliary accumulation of GLI2; while higher FGF signaling is likely due to (1) elevated *Fgfr3* expression in the proliferative zone as a result of compromised Hedgehog signaling, and (2) elevated *Fgf18* expression in the perichondrium. Thus, in a chondrocyte-specific deletion of *Evc2*, we would expect to exclude the impact of increased expression of *Fgf18* in the perichondrium. Aggrecan enhancer-driven, tetracycline-inducible Cre (*ATC*) is a transgenic allele containing an *A**ggrecan* gene enhancer with internal tetracycline regulatory elements that allows Tetracycline-dependent expression of *C**re* recombinase specifically in growth plate and other chondrocytes, but not in the perichondrium [43] (S7B Fig). To specifically delete *Evc2* in chondrocytes we generated ATC; *Evc2* floxed mice. Examination of E18.5 bone showed a significant decrease in *Gli1* and *Pthrp* expression in growth plate chondrocytes (Fig 6G and 6K), indicating decreased Hedgehog signaling in these cells. At the same time, we detected no change in *Spry3* expression (readout for FGF signaling) in the growth plate (Fig 6I) and no change in *Fgf18* expression in the perichondrium (Fig 6J), indicating that neither *Fgf18* expression nor FGF signaling was altered, despite elevated *Fgfr3* expression (Fig 6H). *In situ* results for *Gli1* and *Spry3* also support the notions of compromised Hedgehog signaling while no difference for FGF signaling in tibia at E18.5 (S8 Fig). These *Evc2* conditional mutants showed only a moderate decrease in tibia length (Fig 6D) compared to *Evc2* germ line mutants (Fig 6A and 6B). At E18.5, these mutants only displayed a 10% decrease in the total length of tibiae (Fig 6C and 6D), 20% decrease in the length of the hypertrophic chondrocyte zone (Fig 6C and 6D), 20% decrease in the number of hypertrophic chondrocytes (Fig 6C and 6D), and a 13% decrease in the length of the proliferating chondrocyte zone (Fig 6C and 6D), but no difference in the total length of the growth plate (Fig 6C and 6D).

### Elevated FGF signaling critically contributes to the pathogenesis of dwarfism in *Evc2* mutants

To further demonstrate that *Evc2* loss of function in the perichondrium is critically involved in the elevation of FGF signaling and pathogenesis of dwarfism, we used the *Dermo1(Twist2)*^*Cre*^ allele [44] that demonstrates recombination in both chondrocytes and the perichondrium (S7A Fig). In *Evc2* floxed mice carrying *Dermo1*^*Cre*^, we detected a significant decrease in *Gli1* and *Pthrp* expression (Fig 6L and 6P) and an increase in *Fgfr3* expression (Fig 6M) in the growth plate, as expected. At the same time, we detected increased *Spry3* expression in the growth plate (Fig 6N) and increased *Fgf18* expression in the perichondrium (Fig 6O), indicating elevated FGF signaling. We also detected a dramatic decrease of tibia length (Fig 6E and 6F), which was similar to that observed in *Evc2* germ-line mutants (Fig 6A and 6B). More specifically, at E18.5, we detected a 21% decrease in the overall length of tibiae (Fig 6E and 6F), 52% decrease in the length of the hypertrophic chondrocyte zone (Fig 6E and 6F), 44% decrease in the number of hypertrophic chondrocytes (Fig 6E and 6F), 36% decrease in the length of the proliferating chondrocyte zone (Fig 6E and 6F), and a 21% decrease in the total length of the growth plate (Fig 6E and 6F). Taken together, these data demonstrate that compromised Hedgehog signaling mediated by *Evc2* mutation only partially contributes to the dwarfism in *Evc2* mutants; additionally, elevated FGF signaling, mediated by *Evc2* mutation in the perichondrium, plays a critical role in the pathogenesis of dwarfism in *Evc2* mutants.

### Dwarfism in *Evc2* mutant mice is partially rescued by loss of one copy of *Fgf18*

**Fig 7:**
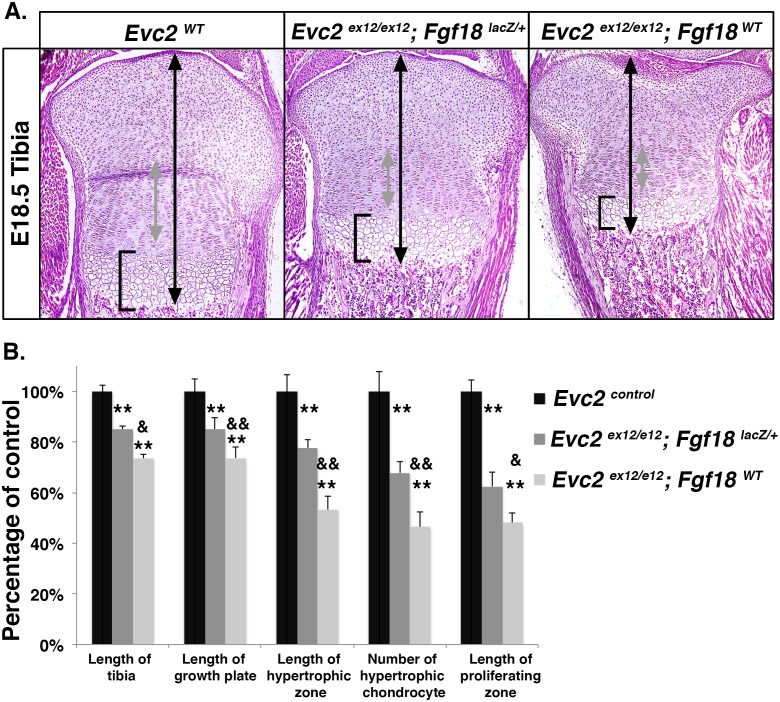
Dwarfism in *Evc2* mutant mice is partially rescued by loss of one copy of *Fgf18*. **A.** H&E staining of tibia proximal growth plates from E18.5 *Evc2* ^*ex12/ex12*^mutants, *Evc2* ^*ex12/ex12*^mutants that are heterozygous for *Fgf18* (*Evc2* ^*ex12/ex12*^; *Fgf18* ^*lacZ/+*^) and littermate controls. B. Quantification of growth plate zone lengths from similar samples as in panel A (n = 6, ** comparing to *Evc2* control, p<0.01; &, comparing to *Evc2* ^*ex12/ex12*^; *Fgf18* ^*LacZ/+*^, p<0.05; &&, comparing to *Evc2* ^*ex12/ex12*^; *Fgf18* ^*LacZ/+*^, p<0.01).

To further demonstrate that elevated FGF signaling plays critical roles during the pathogenesis of dwarfism in *Evc2* mutant mice, we inactivated one allele of *Fgf18* in *Evc2* mutant mice (*Evc2* ^*ex12/ex12*^; *Fgf18* ^*LacZ/+*^). Compared to *Evc2* ^*ex12/ex12*^ mutants, removal of one copy of *Fgf18* allele partially rescued the dwarfism in the *Evc2* ^*ex12/ex12*^; *Fgf18* ^*LacZ/+*^mutant (Fig 7A). More specifically, the length of *Evc2* mutant tibia is 75% of control, while the length of *Evc2* ^*ex12/ex12*^; *Fgf18* ^*LacZ/+*^ tibia is about 82% of control (Fig 7B, n = 6, p<0.05). Similarly, the length of the growth plate, length of the hypertrophic zone, number of hypertrophic chondrocytes and the length of the proliferating zones observed in *Evc2* ^*ex12/ex12*^ mutant tibia are all partially rescued in *Evc2* ^*ex12/ex12*^; *Fgf18*^*LacZ/+*^ embryos. We did not see overt differences between wild type and *Fgf18* heterozygous mutants as previously reported [40]. These results demonstrate that elevated FGF signaling mediated by elevated *Fgf18* expression in the perichondrium critically contributes to the pathogenesis of dwarfism in *Evc2* mutant mice.

## Discussion

The EvC syndrome is categorized as a ciliopathy due to the ciliary localization of the proteins encoded by two causative genes, *EVC* and *EVC2*. The identification of a function for EVC/EVC2 in transducing Hedgehog signaling has classified the pathogenic reason for EvC syndrome as defective Hedgehog signaling through aberrant Hedgehog-PTHrP feedback loop in the growth plate. Here, through genetic approaches, we demonstrate that compromised Hedgehog-PTHrP feedback loop mediated by *Evc2* mutations only partially contributes to the dwarfism; compromised Hedgehog-PTHrP feedback loop is not sufficient in itself to impact growth plate function as severely as observed in *Evc2* germ-line knockout mice. We have additionally demonstrated that elevation of FGF signaling in growth plates due to loss of *Evc2* function in the perichondrium plays a critical role in the pathogenesis of dwarfism in *Evc2* mutants. Overall, our developmental studies provide direct *in vivo* evidence for the function of EVC/EVC2 in transducing Hedgehog signaling; and more importantly, our genetic approaches to investigate the dwarfism in *Evc2* mutant mice uncover a novel pathogenic mechanism to understand limb dwarfism in patients with Ellis-van Creveld syndrome.

### *Evc2* deletion of exon12 abrogates the ciliary function of EVC2

In this work, *Evc2* mutant mice were generated that mimic one of nonsense mutations identified in EvC human patients. Although the mutation is different from that of previously reported *Evc2* mutant mouse line [23], in which *Evc2* was deleted from exon 1, our strategy too resulted in abrogation of EVC2 protein in cilia. Using an antibody recognizing the N-terminus of EVC2, we could not detect any ciliary EVC2 (S2A and S2B Fig) [27], and as a result, EVC also lost its ciliary localization [27]. This observation is consistent with previous studies that deletion of 83 amino acids or more in the C terminus of EVC2 leads to complete loss of ciliary localization [21, 23]. Thus, in our *Evc2* mutant, the ciliary function of the EVC/EVC2 complex is completely abolished, just as it is when *Evc2* is deleted from exon1. Consistently, the length of each appendicular bone is reduced in our *Evc2* mutant mice as much as in the previously reported mutants [23].

### Compromised but not abrogated Hedgehog signaling is consistent with the clinical features of EvC and the phenotypes in *Evc2* mutant mice

Previous *in vitro* biochemical and molecular biological studies indicated that the EVC/EVC2 complex is essential during Hedgehog signaling [21, 23]. However, congenital defects due to abnormally diminished Hedgehog signaling, such as neural tube and midline defects, are not present in EvC patients or in *Evc*/*Evc2* mutant mice. On the other hand, our *in vivo* and *in vitro* studies suggest that an inactivating mutation of *Evc2* leads to compromised but not abrogated Hedgehog signaling. Therefore, we favor the idea that compromised but not abrogated Hedgehog signaling in *Evc2* mutant embryos leads to limited aspects of developmental abnormalities in the processes that require Hedgehog signaling. Similarly, although decreased Hedgehog signaling in the limb bud was detected using a *Ptch1-lacZ* reporter, there was no digit pattern defect in *Evc2* mutant mice [23]. During limb development, loss of function of Indian Hedgehog leads to extremely short limbs and distorted growth plates [13], which are apparently more severe than the phenotypes found in *Evc* [26] and *Evc2* mutant mice ([23] and this work). Chondrocyte-specific postnatal knockout of Indian hedgehog leads to early closure of growth plates, as a result of diminished *Pthrp* expression from as early as postnatal day 15 [31]. Similarly, diminished Hedgehog signaling caused by ablation of primary cilia specifically in cartilage also leads to early closure of growth plate by P15 [45], which is observed in neither *Evc* nor *Evc2* mutant mice for up to 6 weeks [26]. All these facts strongly suggest that in *Evc* and *Evc2* mutant mice there is still a substantial level of Hedgehog signaling remaining. This notion is supported by previous observations that there is still substantial level of *Gli1* expression detectable in *Evc* or *Evc2* mutant growth plates [23, 26]. These phenotypic observations coincide with our finding that *Evc2* mutant primary chondrocytes from embryonic limbs and ribs still retain 40% to 60% of wild type levels of Hedgehog signaling (Fig 2B).

### Elevated FGF signaling is mainly due to elevated *Fgf18* expression in the perichondrium

Since FGF signaling was shown to regulate ciliary length [46], no studies have yet addressed how primary cilia regulate cellular responses to FGF ligands. In primary chondrocytes and MEFs, we did not detect differential responses of control and *Evc2* mutant cells to FGF ligands. On the other hand, we detected both elevated *Fgfr3* expression (Fig 4C) in the growth plate as well as elevated *Fgf18* expression in the perichondrium of *Evc2* mutants (Fig 4D, 4E and 4F). Previous reports [15, 37] and our current work suggest that PTHrP negatively regulates *Fgfr3* expression (S6 Fig). Therefore, elevated *Fgfr3* expression in the growth plate is possibly due to decreased *Pthrp* expression caused by compromised Hedgehog signaling. However, elevated *Fgfr3* expression itself appears insufficient to elevate FGF signaling, since the chondrocyte-specific deletion of *Evc2* that we achieved using *ATC* resulted in compromised Hedgehog signaling and elevated *Fgfr3* expression (Fig 6H), but neither an elevation of *Fgf18* expression in the perichondrium (Fig 6J) nor in an increase in FGF signaling (Fig 6I). Therefore, elevated *Fgf18* expression in the perichondrium likely plays a major role in upregulating FGF signaling in the *Evc2* mutant growth plate. It is still possible that elevated *Fgfr3* has an additive impact in the presence of elevated *Fgf18* expression on the final outcome of FGF signaling. Our work also indicated that elevated expression of *Fgf18* in the perichondrium was a consequence of the *Evc2* mutation in the perichondrium, since elevation of *Fgf18* expression was dependent upon *Evc2* deletion in these cells (Fig 6J and 6O). The molecular mechanism of how loss of *Evc2* in the perichondrium leads to elevated *Fgf18* expression is currently under investigation.

### Elevated FGF signaling is critically important for the pathogenesis of dwarfism in *Evc2* mutants

*ATC*-dependent *Evc2* conditional mutants allowed us to exclude the impact of FGF signaling and thereby to evaluate how a compromised Hedgehog-PTHrP feedback loop results in dwarfism. Despite a mild decrease in the length of their tibiae (Fig 6C and 6D), these mutants had growth plate defects quite different from those of *Evc2* germ-line mutants (Fig 6A and 6B). These results clearly demonstrate that the affected Hedgehog-PTHrP feedback loop mediated by *Evc2* mutation in chondrocytes is not sufficient to impact the length of proliferating chondrocyte zone, hypertrophic chondrocyte zone and growth plate as severely as observed in *Evc2* germ-line mutants (Fig 6A and 6B). In support of this idea, *Dermo1*^*Cre*^-dependent *Evc2* conditional mutants exhibited a more dramatic decrease in tibia length (Fig 6E and 6F) and their growth plate phenotype was similar to that of *Evc2* germ-line mutants. Therefore, both elevated FGF signaling and a compromised Hedgehog-PTHrP feedback loop likely contribute critically to the pathogenesis of dwarfism in *Evc2* mutants. Consistent with our findings, decreased proliferating and hypertrophic chondrocyte zones were also observed in *Evc* mutant mice [26], suggesting a shared mechanism leading to dwarfism in *Evc* and *Evc2* mutant mice.

In conclusion, the results obtained from *in vitro* and *in vivo* suggest a model wherein *Evc2* inactivating mutations as well as *EvC* syndrome causing mutations partially compromise but do not abrogate Hedgehog signaling. The resulting compromised Hedgehog-PTHrP feedback loop only partially contributes to the dwarfism. In addition, our new findings have exposed a novel regulatory mechanism in which *Evc*/*Evc2* inactivating mutations in the perichondrium leads to a significant elevation of FGF signaling. Both effects, i.e., compromised Hedgehog-PTHrP feedback loop and elevated FGF signaling, likely synergize to render the severe dwarfism that characterizes the EvC syndrome. This model thus also suggests that the available therapeutic solutions being tested for Achondroplasia could be used to relieve, at least partially, the severity of dwarfism in EvC patients.

## Materials and Methods

### Animals

The generation of *Evc2* mutant mice and *Evc2* floxed mice was reported elsewhere [27]. *Fgf18*^*LacZ*^ mutant mice were reported previously [40]. *Fgf18*^*GFP*:*CreER*^ mice contain a splice acceptor (SA) GFP:CreERT2 insertion into the first intron of *Fgf18* (D.M.O., I.H.H. unpublished). To obtain *Evc2* homozygous mutant embryos, timed mating between two heterozygous *Evc2* germ-line knockout mice was carried out. Noon of the date when the vaginal plug was observed was designated embryonic day 0.5 (E0.5). *Evc2* floxed mice were bred with mice carrying doxycycline inducible Aggrecan enhancer-driven, tetracycline-inducible Cre (*ATC*)[43] or *Dermo1*^*Cre*^ [44] mice to generate conditional deletions of *Evc2*. For chondrocyte-specific deletion mediated by *ATC*, doxycycline-supplemented chow diet (Harlan TD01306) was provided to pregnant females from E9.5. All mouse experiments were performed in accordance with University of Michigan guidelines and federal laws covering the humane care and use of animals in research. All animal procedures used in this study were approved by the Institutional Animal Care and Use Committee (IACUC) at the University of Michigan (Protocol #PRO00005716).

### Histology, skeletal staining, and immunohistochemistry

Limbs were dissected out from embryos, fixed in 4% paraformaldehyde (PFA), embedded in paraffin, sectioned and stained with hematoxylin and eosin (H&E) according to standard procedures. For skeletal staining, dissected limbs were skinned and stained with alcian blue and alizarin red according to [47, 48]. For immunohistochemistry, limbs were fixed in 4% PFA overnight at 4°C and cryo-protected in 30% sucrose in PBS solution before embedding in OCT. Specimens were cut into 10-μm sections and incubated overnight at 4°C with antibody against EVC2 (Y20, 1:50, Santa Cruz Biotechnology, Santa Cruz, CA, USA), GLI2 (H300, 1:50, Santa Cruz Biotechnology, Santa Cruz, CA, USA), SMO (N19, 1:50, Santa Cruz Biotechnology, Santa Cruz, CA, USA), SUFU (H300, 1:50, Santa Cruz Biotechnology, Santa Cruz, CA, USA), EVC (HPA008703, 1:50, Sigma, St. Louis, MO, USA), KIF7 (Ab95884, 1:100, Cambridge, MA, USA), acetylated tubulin (T6793, 1:1000, Sigma, St. Louis, MO, USA), gamma tubulin (T5326, 1:1000, Sigma, St. Louis, MO), pERK (4695, 1:50, Cell signaling, Danvers, MA 01923), or STAT1 (Ab3987, 1:100, Cambridge, MA, USA). Sections were then incubated with corresponding Alexa Fluor 488 conjugated secondary antibody for 1 h at room temperature before mounting with ProLong Gold Anti-fade Reagent with DAPI (P36935, Life Technologies, Grand Island, NY, USA). All fluorescence images were acquired at room temperature by confocal microscopy (Nikon C1) through Nikon EZ-C1 3.91 and processed by Adobe Photoshop CS6.

### Isolation of primary chondrocytes and immunocytochemistry

For primary chondrocyte isolation, rib or long bone cartilage was dissected from E18.5 embryos and digested with collagenase A (Roche, Indianapolis, IN, USA). Chondrocytes released will be subsequently cultured in DMEM (Life Technology, Grand Island, NY, USA) with 10% FBS (Atlanta Biologicals, Flowery Branch, GA, USA). Experiment will be carried out using cells within 5 passages. For immunocytochemistry, cultured primary chondrocytes were starved in 0.5% serum for 36 h before treatment with 100 nmol of SAG (Chemicon, Billerica, MA, USA) for 4 h. Cells were then fixed in 4% PFA and permeabilized in PBS with 0.1% Triton X-100 (Sigma, St. Louis, MO, USA) before incubation with primary antibody at 4°C for overnight and with fluorescent secondary antibody. Mounting was done with ProLong Gold Anti-fade Reagent containing DAPI.

### RNA isolation and quantitative real-time PCR

RNA was isolated from primary chondrocytes using TRIzol (Life Technologies, Grand Island, NY, USA) according to manufacturer’s instructions. For RNA isolation from embryonic growth plates, long bones were dissected out at E16.5. Growth plates were collected from tibiae and placed into TRIzol for homogenization according to manufacturer’s instructions. For isolation of perichondrium cells, growth plates were dissected out from embryonic tibiae and digested with 1 unit /ml Dispase [48]. For reverse transcription, 1 μg of total RNA was reverse-transcribed using SuperScript Reverse Transcriptase (Life Technologies, Grand Island, NY, USA). Quantitative real-time PCR was performed using Applied Biosystems ViiA7, with the following taqman probes: Mm00494645_m1 for *Gli1*, Mm99999915_g1 for *Gapdh*, Mm00439612_m1 for *Ihh*, Mm00436026_m1 for *Ptch1*, Mm00436057_m1 for *Pthrp*, Mm00433294_m1 for *Fgfr3*, Mm00438941_m1 for *Fgfr2*, Mm00438932_m1 for *Fgfr1*, Mm00432448_m1 for *Cdkn1a* (P21), and Mm00433286_m1 for *Fgf18*.

### Tibia *ex-vivo* culture

Tibiae were dissected out from hindlimbs of E16.5 embryos and placed into 24-well plates with 1 ml medium (alpha MEM (Life Technologies, Grand Island, NY, USA), 0.5% FBS, penicillin and streptomycin (Life Technologies, Grand Island, NY, USA). Media were changed every other day, and the full length of tibia was measured at day 1 (D1) and D7. The ratios of the lengths at D7 over the lengths at D1 was calculated and compared between controls and mutants.

### RNA *in situ* hybridization

RNA in situ hybridization was carried out as previously described [49] using a digoxygenin-labeled *Fgf18* probe [39, 40]. Briefly, embryonic tissues were immediately fixed in 4% PFA, before cryo-protected in 30% sucrose in PBS. Then, 20 μm sections were treated with proteinase K, post-fixed with 4% PFA, before treated with acetic anhydride solution (Sigma, St. Louis, MO, USA). Sectioned tissues were hybridized with RNA probe in hybridization solution containing 5X SSC, 50% formamide, 1mg/mg tRNA (Sigma, St. Louis, MO, USA), 0.1mg/ml Heparin (Sigma, St. Louis, MO, USA) at 65°C. Sectioned tissues were then digested with RNase A (Roche) and washed in post-hybridization washing solution containing 0.2X SSC, before incubation with alkaline phosphatase conjugated mouse anti-digoxygenin for overnight. Purple color for positive signal was developed through incubation sections with BM Purple for AP substrate precipitating (Roche).

## Supporting Information

S1 Fig Generation of *Evc2* mutant mice. A. Diagram showing that the Evc2 mutant allele was obtained by inserting a premature stop codon and IRES-lacZ cassette into exon 12. B. Lateral views of an *Evc2* mutant and a littermate control at 4 weeks of age. C-D. Quantification of body length (C) and body weight (D) at E18.5. Data are presented as percentages of controls (n = 10, p>0.2). E. X-gal staining of the tibia proximal growth plate of *Evc2* wild-type (*Evc2* ^*WT*^) and heterozygous mutant (*Evc2* ^*+/ex12*^) littermates indicates Evc2 expression in growth plate chondrocytes, perichondrocytes and other neighbor tissue cells. Scale bars: 200 μm for (D).
(TIF)

S2 Fig Loss of EVC2 in the cilia of *Evc2* mutant chondrocytes. Immunohistochemistry with EVC2 and acetylated tubulin antibodies allow visualization of EVC2 at the tip of cilia in humerus growth plate chondrocytes in E15.5 control animals (A), but not in mutant littermates (B). Scale bars: 200 μm for (A) and (B).
(TIF)

S3 Fig Analysis of gene expression in *Evc2* mutant growth plate. *In situ* hybridization of *Ihh* (A), *ColII* (B), and *ColX* (C) in E18.5 distal ulna and radii.
(TIF)

S4 Fig Analysis of cilia markers in *Evc2* mutant cells. Primary chondrocytes from control and *Evc2* mutant littermates were treated with 100 nM SAG for 8 h and subjected to immunocytochemistry for acetylated tubulin and GLI2 (A), SUFU (C), KIF7 (E) and SMO (G). The percentages of cilia positive for the indicated proteins are shown in B, D, F and H (n = 80). Scale bars: 10 μm for (A), (B), (C) and (D).
(TIF)

S5 Fig *Evc2* mutant growth plate has elevated FGF signaling. In situ hybridization of *Spry3* (A) and *Fgfr3* (B) in E18.5 proximal tibia.
(TIF)

S6 Fig *Fgfr3* in vitro analysis. qRT-PCR assay of *Fgfr3* mRNA levels in primary chondrocytes with no treatment or treated with PTH for 24 h (n = 3, **p<0.01). Data are presented as percentages of untreated controls.
(TIF)

S7 Fig Verification of the recombination activity of the *Dermo1-Cre* and *ATC* transgenes. A. X-gal staining of proximal tibiae from E17.5 or E18.5 embryos carrying *Dermo1*^*Cre*^ (A) or *ATC* (B) and a Cre-dependent *ROSA26R*^*LacZ*^ allele demonstrates that *ROSA26R*^*LacZ*^ is efficiently recombined in both chondrocytes and perichondrium of embryos carrying *Dermo1*^*Cre*^, but that it is recombined only in chondrocytes in embryos carrying *ATC*. C. Diagram showing the generation of *Evc2* floxed mutant mice. LoxP sites were inserted into mouse *Evc2* to flank exon13 and exon14. Scale bars: 200 μm for (A) and (B).
(TIF)

S8 Fig Analysis of gene expression in *Evc2* ATC conditional mutant growth plate. In situ hybridization of *Gli1* (A) and *Fgfr3* (B) in E18.5 proximal tibia in growth plates from *Evc2 ATC* conditional mutant and littermate controls.
(TIF)
