# Supplementary figures and images for: Elevated Fibroblast Growth Factor Signaling Is Critical for the Pathogenesis of the Dwarfism in Evc2/Limbin Mutant Mice
Source: PLoS Genet. 2016 Dec 27;12(12):e1006510. doi: 10.1371/journal.pgen.1006510 (PMC5189957; doi:10.1371/journal.pgen.1006510)

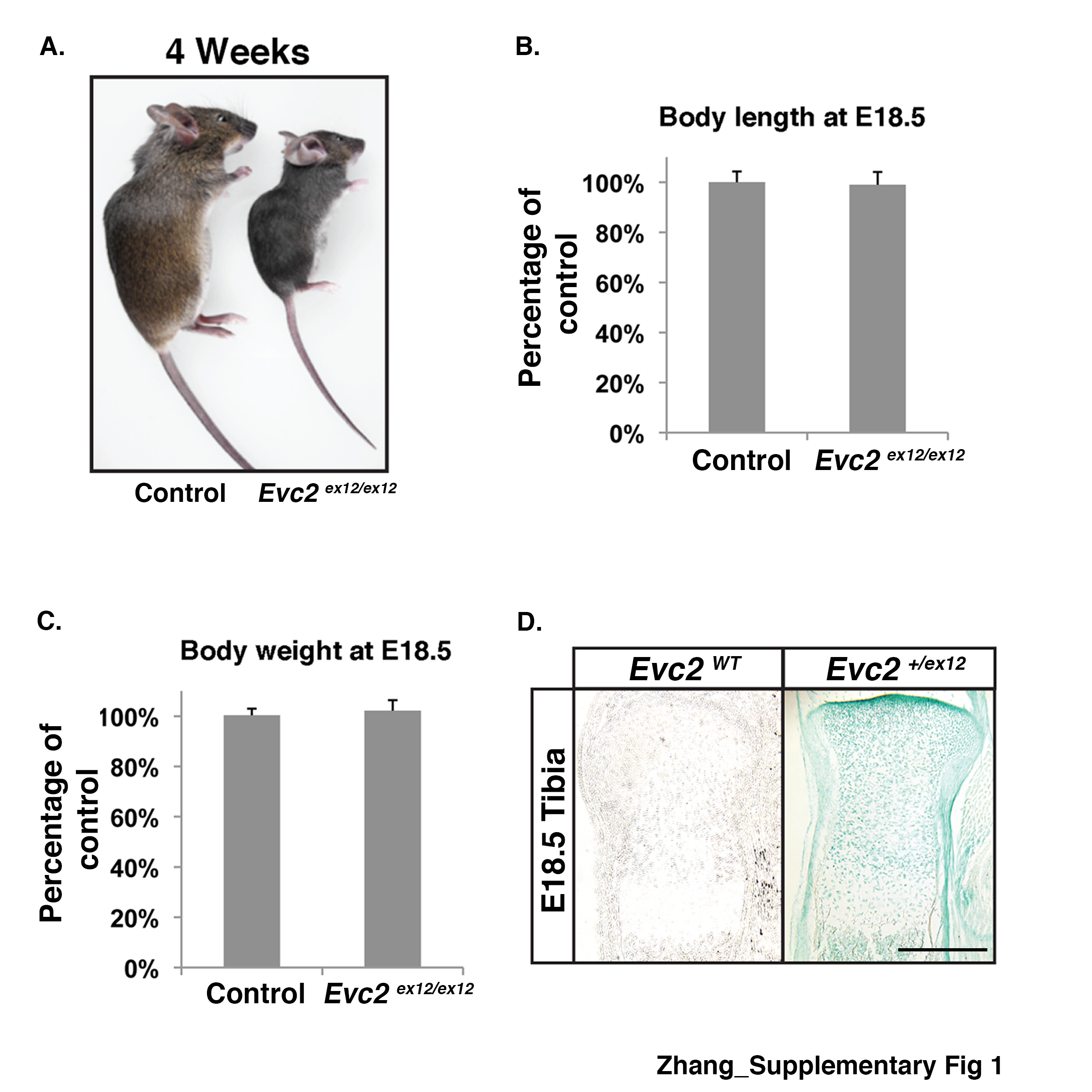

Supplement: S1 Fig — A. Diagram showing that the Evc2 mutant allele was obtained by inserting a premature stop codon and IRES-lacZ cassette into exon 12. B. Lateral views of an Evc2 mutant and a littermate control at 4 weeks of age. C-D. Quantification of body length (C) and body weight (D) at E18.5. Data are presented as percentages of controls (n = 10, p>0.2). E. X-gal staining of the tibia proximal growth plate of Evc2 wild-type (Evc2 WT) and heterozygous mutant (Evc2 +/ex12) littermates indicates Evc2 expression in growth plate chondrocytes, perichondrocytes and other neighbor tissue cells. Scale bars: 200 μm for (D). (TIF) [file pgen.1006510.s001.tif]

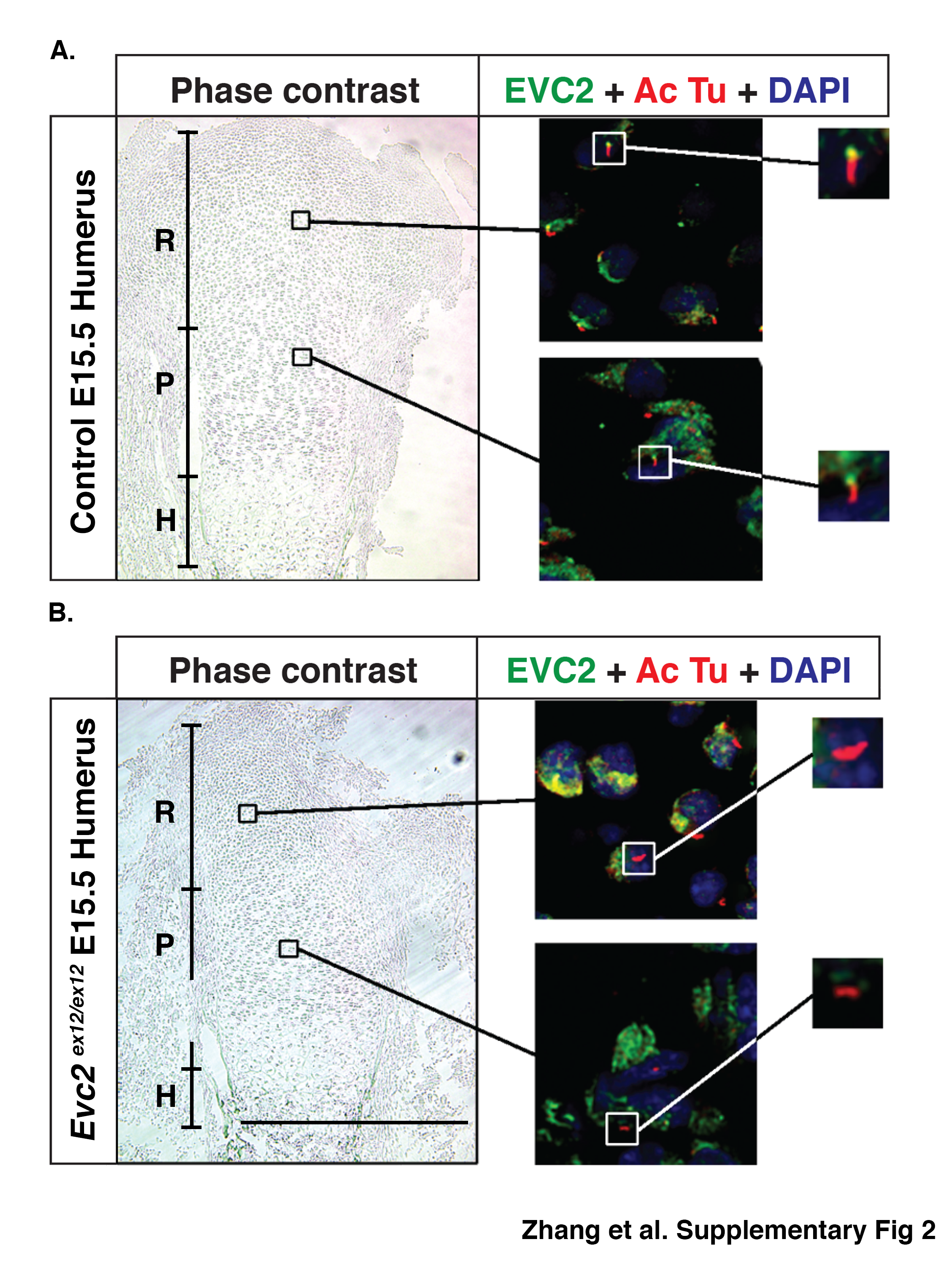

Supplement: S2 Fig — Immunohistochemistry with EVC2 and acetylated tubulin antibodies allow visualization of EVC2 at the tip of cilia in humerus growth plate chondrocytes in E15.5 control animals (A), but not in mutant littermates (B). Scale bars: 200 μm for (A) and (B). (TIF) [file pgen.1006510.s002.tif]

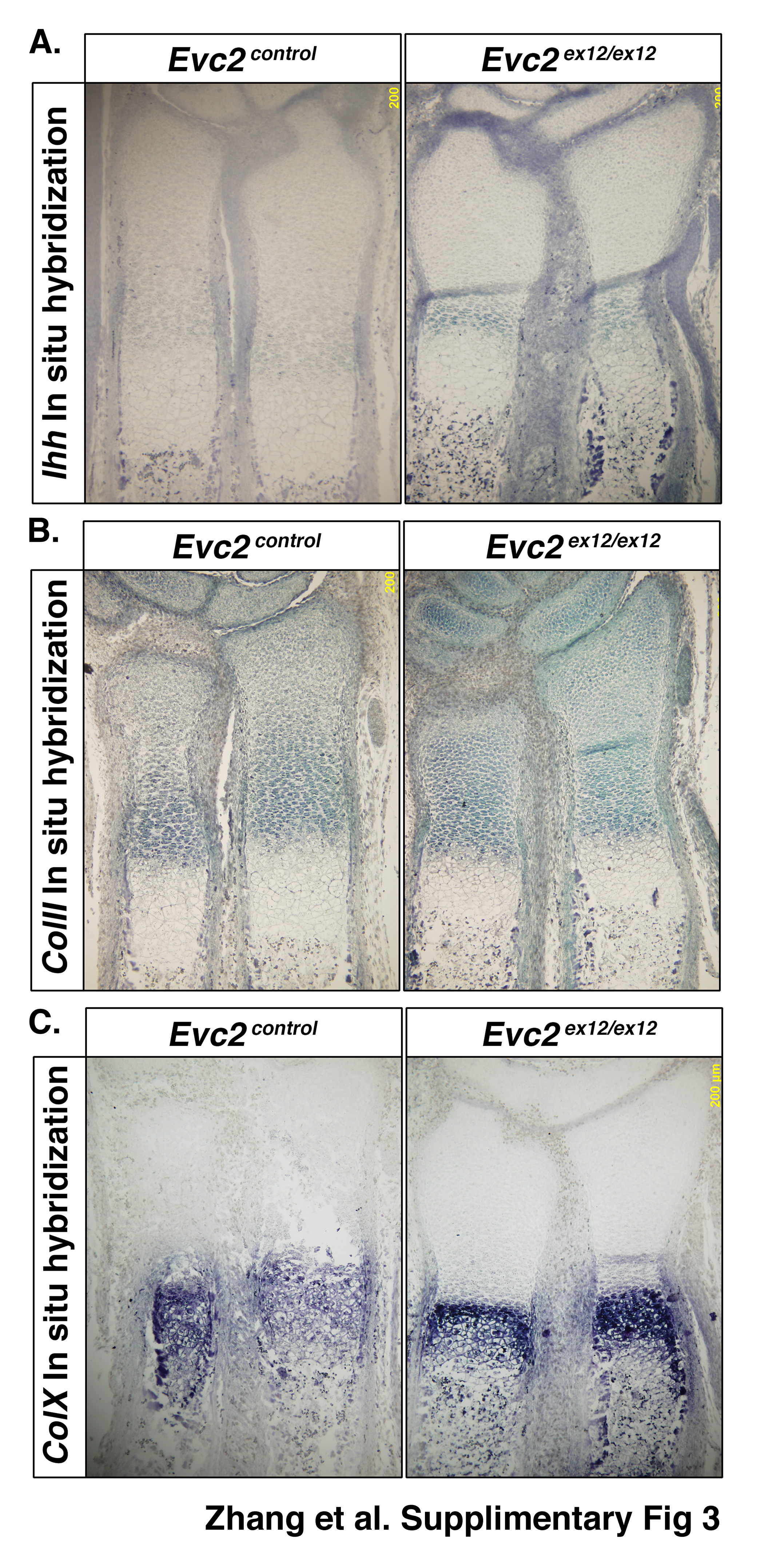

Supplement: S3 Fig — In situ hybridization of Ihh (A), ColII (B), and ColX (C) in E18.5 distal ulna and radii. (TIF) [file pgen.1006510.s003.tif]

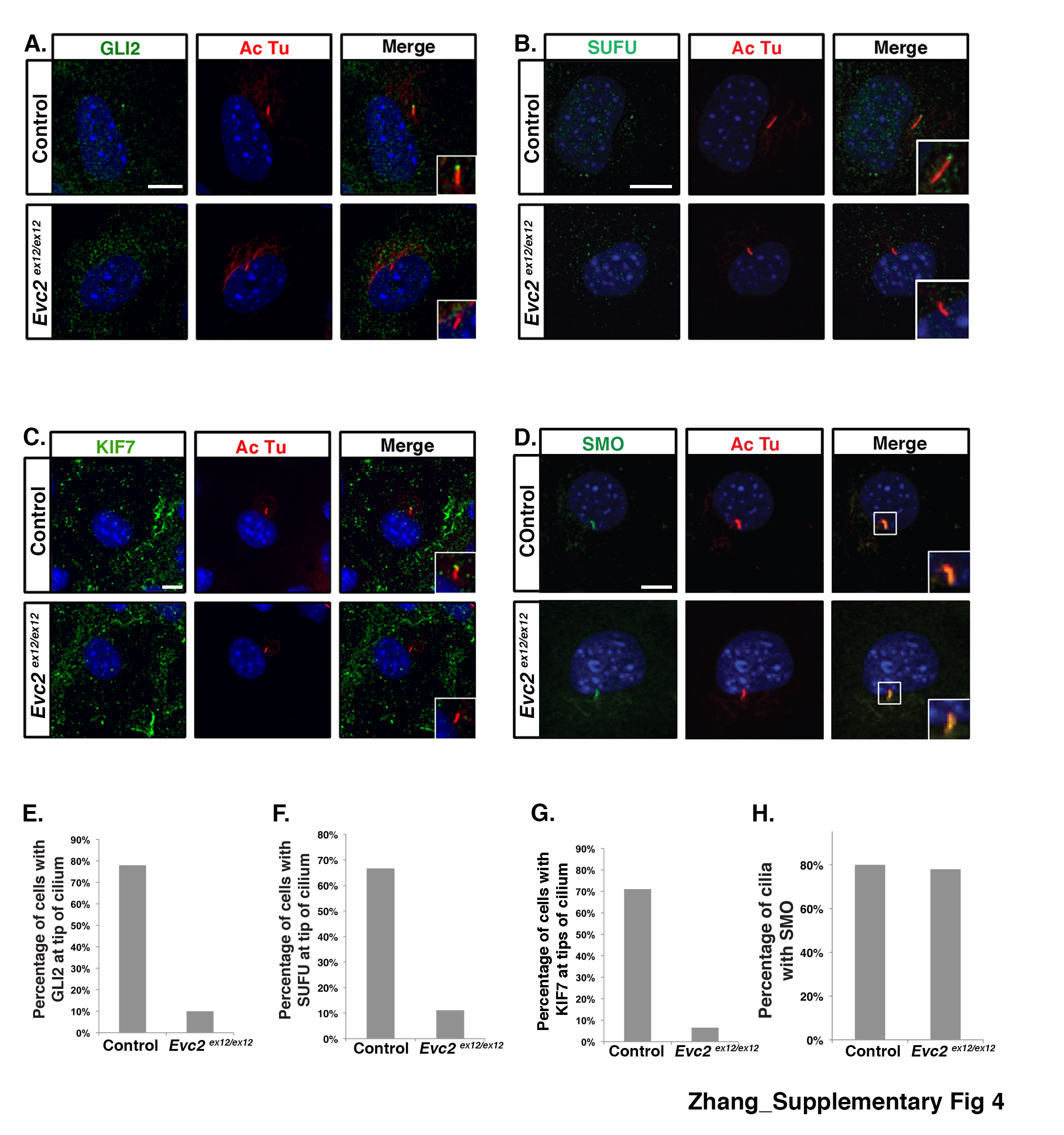

Supplement: S4 Fig — Primary chondrocytes from control and Evc2 mutant littermates were treated with 100 nM SAG for 8 h and subjected to immunocytochemistry for acetylated tubulin and GLI2 (A), SUFU (C), KIF7 (E) and SMO (G). The percentages of cilia positive for the indicated proteins are shown in B, D, F and H (n = 80). Scale bars: 10 μm for (A), (B), (C) and (D). (TIF) [file pgen.1006510.s004.tif]

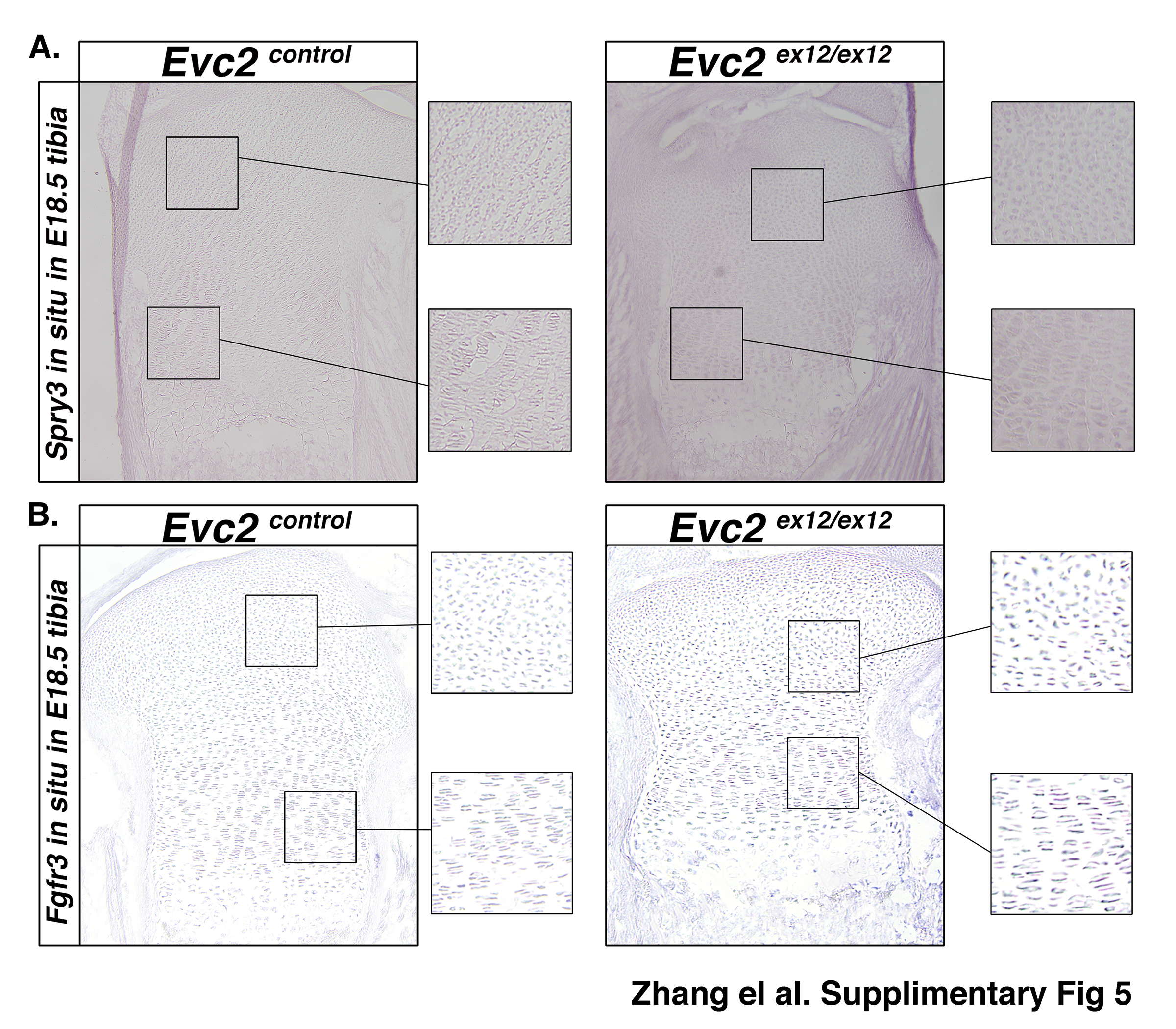

Supplement: S5 Fig — In situ hybridization of Spry3 (A) and Fgfr3 (B) in E18.5 proximal tibia. (TIF) [file pgen.1006510.s005.tif]

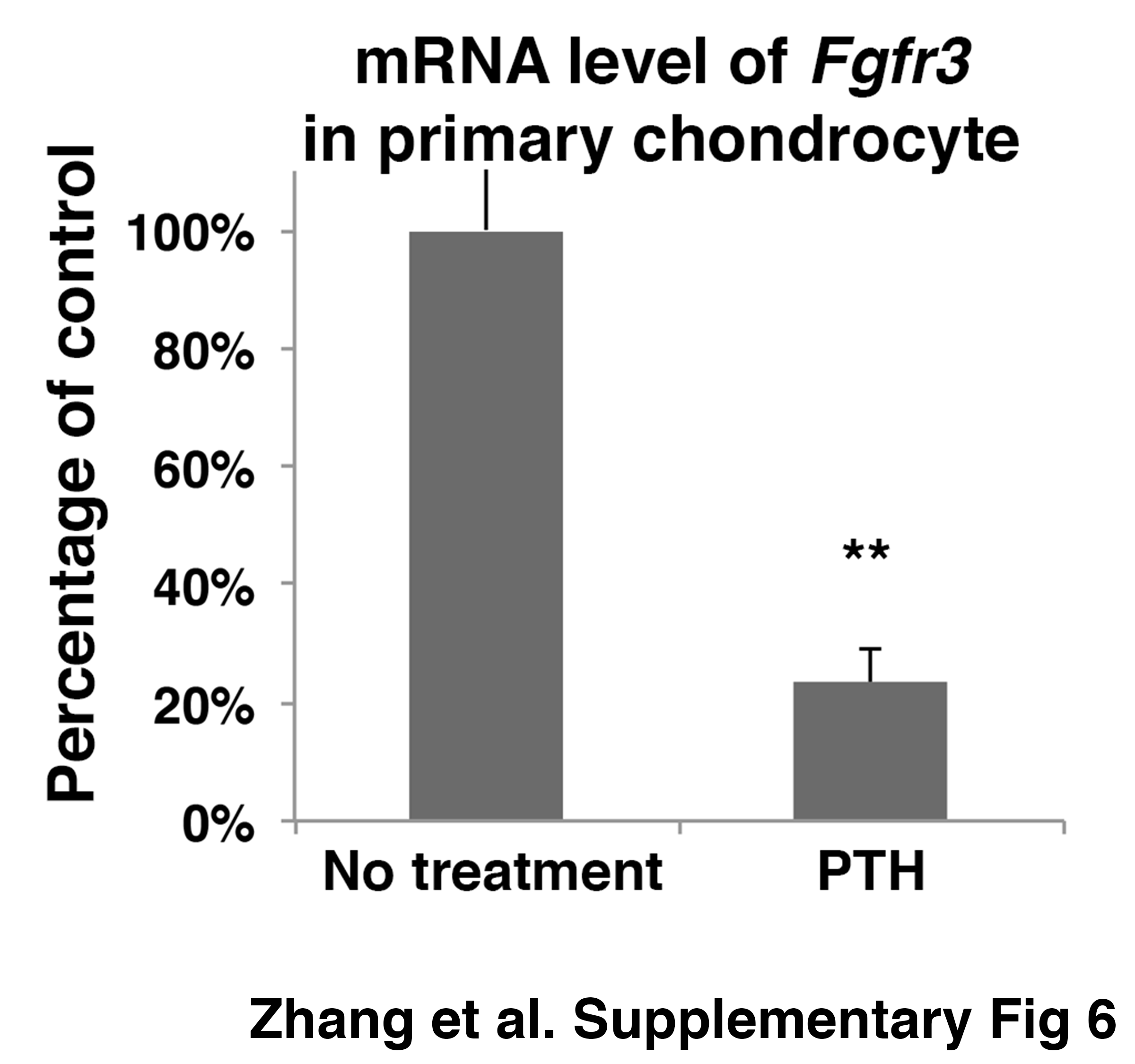

Supplement: S6 Fig — qRT-PCR assay of Fgfr3 mRNA levels in primary chondrocytes with no treatment or treated with PTH for 24 h (n = 3, **p<0.01). Data are presented as percentages of untreated controls. (TIF) [file pgen.1006510.s006.tif]

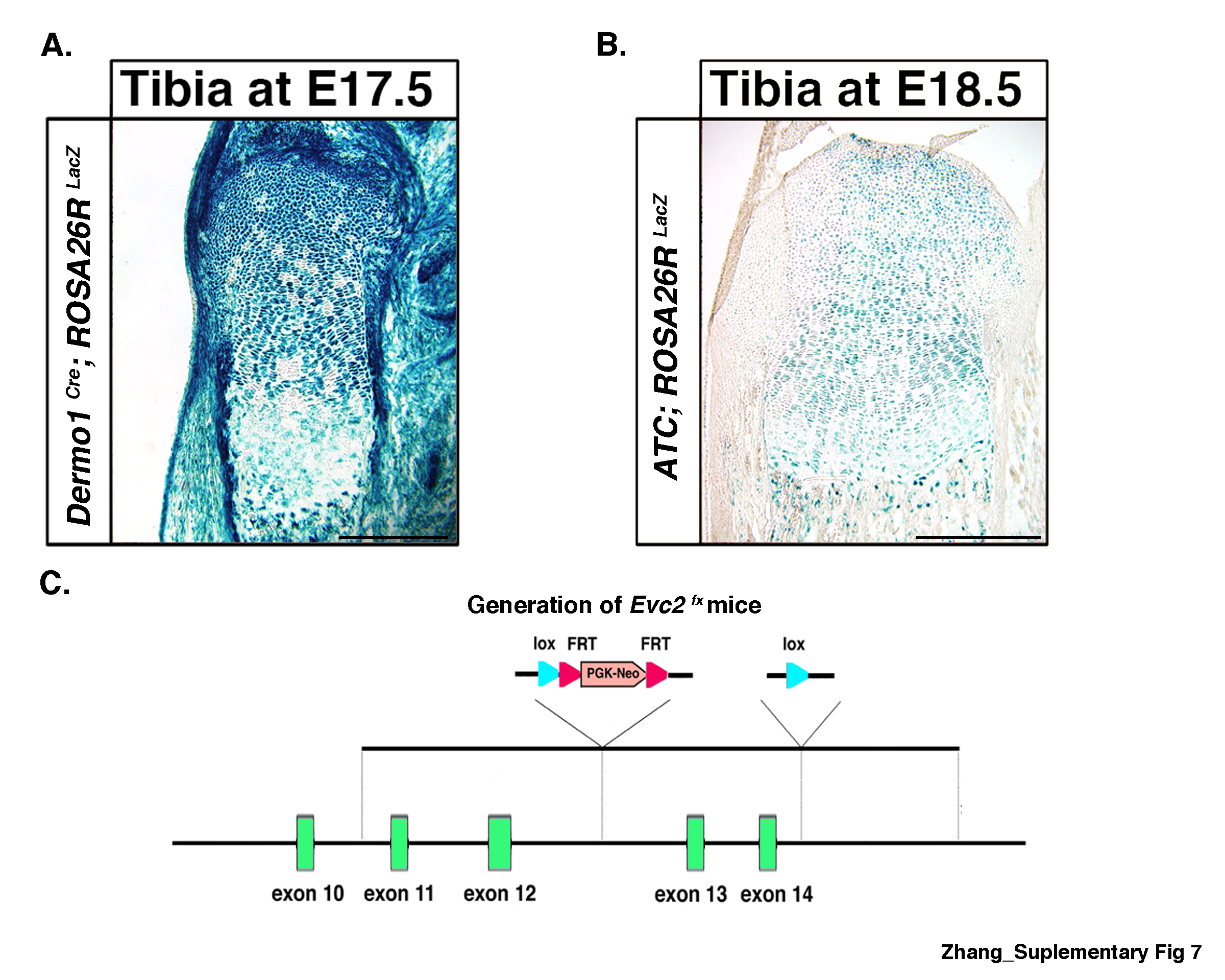

Supplement: S7 Fig — A. X-gal staining of proximal tibiae from E17.5 or E18.5 embryos carrying Dermo1Cre (A) or ATC (B) and a Cre-dependent ROSA26RLacZ allele demonstrates that ROSA26RLacZ is efficiently recombined in both chondrocytes and perichondrium of embryos carrying Dermo1Cre, but that it is recombined only in chondrocytes in embryos carrying ATC. C. Diagram showing the generation of Evc2 floxed mutant mice. LoxP sites were inserted into mouse Evc2 to flank exon13 and exon14. Scale bars: 200 μm for (A) and (B). (TIF) [file pgen.1006510.s007.tif]

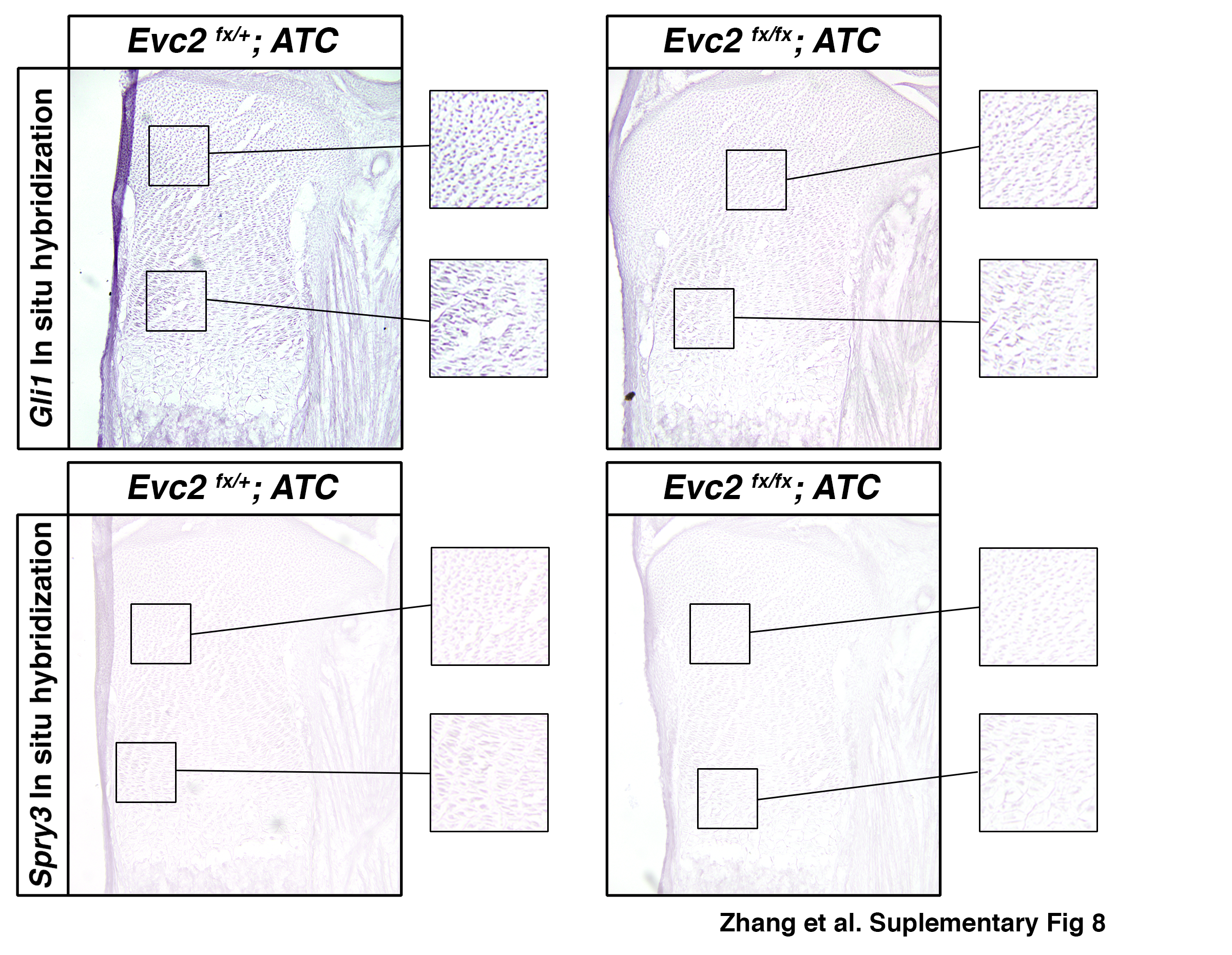

Supplement: S8 Fig — In situ hybridization of Gli1 (A) and Fgfr3 (B) in E18.5 proximal tibia in growth plates from Evc2 ATC conditional mutant and littermate controls. (TIF) [file pgen.1006510.s008.tif]
